# Genistein mitigates diet-induced obesity and metabolic dysfunctions in gonadectomized mice with some sex-differential effects

**DOI:** 10.3389/fendo.2024.1392866

**Published:** 2024-09-16

**Authors:** Weerapat Kositanurit, Natakorn Siritaweechai, Pachara Varachotisate, Chuti Burana, Narittee Sukswai, Jerasit Surintrspanont, Prasong Siriviriyakul, Kasiphak Kaikaew, Duangporn Werawatganon

**Affiliations:** ^1^ Department of Physiology, Faculty of Medicine, Chulalongkorn University, Bangkok, Thailand; ^2^ Precision Pathology of Neoplasia Research Group, Department of Pathology, Faculty of Medicine, Chulalongkorn University, Bangkok, Thailand; ^3^ Department of Pathology, King Chulalongkorn Memorial Hospital, The Thai Red Cross Society, Bangkok, Thailand; ^4^ Center of Excellence in Alternative and Complementary Medicine for Gastrointestinal and Liver Diseases, Chulalongkorn University, Bangkok, Thailand

**Keywords:** high-fat high-sucrose diet, insulin resistance, metabolic dysfunction-associated steatotic liver disease (MASLD), phytoestrogen, sex difference

## Abstract

**Background:**

Obesity is associated with insulin resistance (IR) and metabolic dysfunction-associated steatotic liver disease (MASLD). Genistein, an isoflavone, is a promising natural compound for preventing and treating obesity and metabolic dysfunctions. We aimed to investigate the sex-specific protective effects of genistein on obesity, IR, and MASLD in a murine model of sex hormone deprivation with diet-induced obesity (DIO), mimicking postmenopausal women or aging men with metabolic syndrome.

**Methods:**

Gonadectomized and sham-operated C57BL/6NJcl mice were fed a high-fat high-sucrose diet for 4 weeks to induce obesity (7 mice per group). In gonadectomized mice, genistein (16 mg/kg/day) or vehicle (7.5% dimethyl sulfoxide) was orally administered for 45 days. We assessed glucose homeostasis parameters, hepatic histopathology, and hepatic gene expression to investigate the effects of gonadectomy and genistein treatment.

**Results:**

Gonadectomy exacerbated adiposity in both sexes. Ovariectomy diminished the protective effects of female gonadal hormones on the homeostatic model assessment for insulin resistance (HOMA-IR), serum alanine transaminase levels, hepatic steatosis score, and the expression of hepatic genes associated with MASLD progression and IR, such as *Fasn*, *Srebf1*, *Saa1*, *Cd36*, *Col1a1*, *Pck1*, and *Ppargc1a*. Genistein treatment in gonadectomized mice significantly reduced body weight gain and the hepatic steatosis score in both sexes. However, genistein treatment significantly attenuated HOMA-IR and the expression of the hepatic genes only in female mice.

**Conclusion:**

Genistein treatment mitigates DIO-related MASLD in both male and female gonadectomized mice. Regarding hepatic gene expression associated with MASLD and IR, the beneficial effect of genistein was significantly evident only in female mice. This study suggests a potential alternative application of genistein in individuals with obesity and sex hormone deprivation, yet pending clinical trials.

## Introduction

Obesity is a disease that is related to metabolic dysfunctions, such as insulin resistance (IR) and metabolic dysfunction-associated steatotic liver disease (MASLD). Obesity can affect whole-body glucose and lipid metabolism. The excess in fatty acid synthesis or uptake by the liver can aggravate lipid droplet accumulation in hepatic parenchyma, which is the pathogenesis of MASLD (1, 2). Sex hormone deprivation is also found to be associated with MASLD. In men, previous studies have shown that androgen deficiency increases hepatic steatosis by increasing *de novo* lipogenesis in the liver. In women, a large body of evidence demonstrates that estrogen has both protective and therapeutic effects on MASLD, and estrogen deficiency is associated with obesity and MASLD (3, 4).

Epidemiological studies have demonstrated that women in most ethnic groups have a higher prevalence of obesity and metabolic syndrome than men. However, women of reproductive age exhibit a lower prevalence of metabolic diseases compared to men (5, 6). Additionally, the prevalence of prediabetic syndrome differs among the sexes: impaired fasting glucose is more prevalent in men, whereas impaired glucose tolerance is more prevalent in women (7). This sex difference can be mainly attributed to the effects of gonadal hormones, as endogenous estrogens exert crucial protective effects through estrogen receptors (ERs) in various tissues (7, 8).

Genistein is an isoflavone, a natural phytoestrogen found in high concentrations in legumes, such as soybeans and soy-rich products (9). In target tissues, genistein can bind to ERs, both ERα and ERβ, and exhibits estrogenic or anti-estrogenic activities, acting as a natural selective estrogen receptor modulator (SERM) (9, 10). Genistein has been extensively employed in studying metabolic diseases in animal models, revealing its beneficial effects on obesity, IR, and systemic inflammation (11, 12). However, whether genistein exhibits sex-specific protective effects on obesity and metabolic dysfunctions, particularly IR and MASLD, has not been thoroughly investigated. Given that sex hormones play a crucial role in the progression and occurrence of metabolic dysfunctions, we aimed to study the effects of genistein treatment in gonadectomized male and female mice to eliminate the direct effects of endogenous sex hormones and to mimic the postmenopausal and male-aging states. In this study, obesity and metabolic dysfunctions were induced by a high-fat high-sucrose (HFHS) diet, resembling the global trend of unhealthy diets.

## Materials and methods

### Animals and housing conditions

Seven-week-old C57BL/6NJcl mice (21 males and 21 females) were obtained from Nomura Siam International (Bangkok, Thailand; a local distributor for M-CLEA Bioresource). Upon arrival, mice were group-housed (3–4 mice per cage) at a room temperature of 22 ± 2°C with 50 ± 2% humidity, and on a 12-hour light/12-hour dark cycle. During the one-week acclimatization period, standard rodent chow and water were available ad libitum. The standard chow (cat. no. 082G, Perfect Companion Group, Bangkok, Thailand) consisted of approximately 26% protein, 6% fat, and 53% carbohydrate by weight, providing approximately 3.69 kcal/g, with about 14% calories from fat and 58% calories from carbohydrate.

### Ethics statement

This animal study was reviewed and approved by the Animal Ethics Committee of the Faculty of Medicine, Chulalongkorn University, Bangkok, Thailand (approval number 017/2564). The study was conducted in accordance with the institutional and national requirements, which included the principles of the 3Rs: replacement, reduction, and refinement. The sample size was calculated based on our previous study (13).

### Gonadectomy

After 7 days of acclimatization (at 8 weeks of age), 28 mice (14 males and 14 females) underwent gonadectomy under isoflurane anesthesia and ketorolac analgesia (5 mg/kg, subcutaneous injection). For female mice, small incisions were made in both flanks to remove the ovaries. In male mice, small incisions were made at both scrotums to remove the testes. Bleeding was checked and stopped, and then the muscle layer and skin were sutured. Sham operation was performed on 14 mice (7 males and 7 females) using the same procedure, except for the removal of the gonads. After the operation, mice were observed until fully awake. Routine post-operative care was monitored daily for a week, including food intake, mobility, general physical activity, and surgical wound inspection. The success of ovariectomy (gonadectomy in females) was confirmed by performing vaginal cytology for 5 consecutive days after the surgery. The absence of a regular estrous cycle indicates successful ovariectomy. In contrast, confirming successful orchiectomy (gonadectomy in males) is omittable, given that complete removal of the testes is generally achieved by the standard surgical procedure.

### Experimental protocol

After the one-week recovery phase from gonadectomy (at 9 weeks of age), all mice were switched to a HFHS diet to induce obesity and metabolic dysfunction. The HFHS diet was prepared in-house by mincing the chow pellets, adding lard (50% w/w), and adding sucrose (15% w/w). This formulation closely resembled the commercially available HFHS diet used for the diet-induced obesity (DIO) model (14, 15). The final composition of the HFHS diet included approximately 57% of calories from fat and 31% from carbohydrate, with an energy content of approximately 5.33 kcal/g. The mice were maintained on the HFHS diet for 4 weeks. After this period, 13-week-old mice of each sex were divided into three experimental groups: sham-operated mice treated with vehicle (Sham), gonadectomized mice treated with vehicle (GDX), gonadectomized mice treated with genistein (GDX+Gen).

A vehicle solution was prepared by mixing 7.5% v/v dimethyl sulfoxide (DMSO) in distilled water. Genistein (Cayman Chemical, purity ≥98%, cat. no. 10005167, purchased from Chemical Express Thailand, Samut Prakan, Thailand) was then dissolved in the 7.5% DMSO solution, resulting in a final concentration of 25 mg/mL. Starting from 13 weeks of age, the genistein-treated groups received an oral dose of 16 mg/kg/day, based on our previous studies (13, 16), which was equivalent to a human dose of 1.3 mg/kg/day (17). Both the vehicle and genistein were administered daily, 5 days per week, for a total duration of 45 days.

Body weight (BW) and food intake were measured and recorded weekly from the initiation of the HFHS diet until the end of the experiment. At 21 weeks of age (12 weeks on the HFHS diet), an intraperitoneal glucose tolerance test (IPGTT) was conducted to assess glucose handling capacity. One week after the IPGTT (at 22 weeks of age), mice were euthanized by administering an overdose of isoflurane anesthesia followed by cardiac puncture.

### Intraperitoneal glucose tolerance test

The mice were fasted for 5 hours. After the fasting period, tail clipping was performed. One small drop of blood was used to instantly determine glucose levels using Accu-Check Guide (Roche Diabetes Care, Bangkok, Thailand), and approximately 25 μL of blood was collected in a heparinized capillary tube for further measurement of plasma insulin levels. Subsequently, an intraperitoneal injection of 20% glucose (in sterile water) was administered at a final dose of 2 g of glucose per kg of BW. Blood glucose levels were measured at 15, 30, 60, 90, and 120 minutes after glucose injection. After the last measurement, the HFHS diet was resumed.

### Plasma insulin determination

The heparinized blood was centrifuged at 1,000×g for 10 minutes at 4°C. Plasma was then collected and stored in −80°C until further steps. Plasma insulin levels were determined using the Ultra Sensitive Mouse Insulin ELISA kit (cat. no. 90080, Crystal Chem, obtained from Chemical Express Thailand, Samut Prakan, Thailand), according to the manufacturer’s instructions.

### Endpoint blood and tissue collection

At the end of the experiment (at 22 weeks of age), mice were fasted for 5 hours. Subsequently, blood was obtained through cardiac puncture under an overdose of isoflurane anesthesia. The blood samples were kept on ice until fully clotted, then centrifuged to collect serum, which was stored in −80°C until serum biomarker determination.

The liver and various white adipose tissues (WAT), including gonadal WAT, axillary WAT (so-called anterior subcutaneous WAT), and inguinal WAT (so-called posterior subcutaneous WAT), were dissected and weighed. The liver was divided into 2 pieces: the first half was fixed in 10% formalin in phosphate buffered saline (PBS), while the second half was snap-frozen in liquid nitrogen and stored in −80°C until further processing. Organ weights were calculated and presented as both actual organ weights and the ratio of organ weight to BW. To clarify, the WAT weight refers to the sum weight of the mentioned WAT depots and the liver index refers to the ratio of liver weight to BW.

### Serum alanine transaminase determination

Serum alanine transaminase (ALT) levels were determined using Reflotron GPT (ALT) strips with the Reflotron Plus Clinical Chemistry Analyzer (Roche Diagnostics, Bangkok, Thailand), following the manufacturer’s instructions.

### Histopathology

Formalin-fixed livers were embedded in paraffin, sectioned with a microtome, mounted on glass slides, and stained with hematoxylin and eosin following standard procedures. The histopathology of liver tissues was examined by two pathologists blinded to the experimental groups, and the severity of fat accumulation in the liver was graded as the steatosis score, ranging from 0 to 9 (18). In brief, the score was an unweighted sum of three domains: macrovesicular steatosis, microvesicular steatosis, and hypertrophy. In each domain, the score ranged from 0 to 3, depending on the percentage of the total area of involvement: 0 means less than 5% affected, 1 means 5–33% affected, 2 means 34–66% affected, and 3 means greater than 66% affected. Representative images from each group were captured using a digital imaging system consisting of a Nikon Eclipse E200 upright microscope equipped with a high-definition color camera and a control unit DS-Fi2-L3 (Nikon Instruments, Tokyo, Japan).

### Gene expression quantification

The frozen liver tissues were homogenized, and ribonucleic acid (RNA) was extracted using the GENEzol reagent (Geneaid, New Taipei City, Taiwan). Contaminating genomic deoxyribonucleic acid (DNA) was removed using RQ1 RNase-Free DNase (Promega Corporation, Madison, WI). Purified RNA was quantified with a NanoDrop 2000 spectrophotometer (Thermo Fisher Scientific, Wilmington, DE). Reverse transcription was performed using the iScript Reverse Transcription Supermix, and quantitative PCR was performed using the SsoAdvanced Universal SYBR Green Supermix (both reagents were from Bio-Rad Laboratories, Hercules, CA) with a QuantStudio 6 Flex Real-Time PCR System (Applied Biosystems, Foster City, CA). Expression levels of the genes of interest were normalized to the housekeeping genes, *Rn18s* and *Actb*, using the 2^−ΔΔCT^ method. Sequences of the primers for all genes are presented in **Supplementary Table 1**.

### Statistical analysis

Data were analyzed and graphs were plotted using GraphPad Prism for Windows (version 9, GraphPad Software, Boston, MA). Continuous data were presented as the mean and standard deviation (SD). Differences in a dependent variable influenced by independent variables [sex and intervention (int)] were analyzed by 2-way ANOVA. When appropriate, the Tukey’s multiple comparisons *post hoc* test was performed to assess differences between groups of the same sex to explore the effect of each intervention, i.e., gonadectomy with or without genistein treatment. The Šídák’s multiple comparisons *post hoc* test was performed to assess sex differences between groups of the same intervention: Sham, GDX, or GDX+Gen. Additionally, a stepwise linear regression analysis was conducted to identify significant factors contributing to changes in BW and the liver index for the GDX and GDX+Gen groups using IBM SPSS Statistics (version 29, IBM, Chicago, IL). A *P*-value of <0.05 was considered statistically significant.

When analyzing the IPGTT data, the area under the curve (AUC) of time (min) and glucose level (mg/dL) was calculated. To assess the degree of IR, we calculated the homeostatic model assessment for insulin resistance (HOMA-IR) using the formula:


$$\text{HOMA–IR=}\frac{\text{[insulin]}\times \text{[glucose]}}{{\text{([insulin]}\times \text{[glucose])}}_{\text{male\_Sham}}}$$

In this formula, [insulin] refers to the fasting plasma insulin level, [glucose] refers to the blood glucose level, and the denominator is the average value of the male Sham group. Thus, this HOMA-IR serves as the IR index relative to sham-operated male mice with vehicle treatment.

## Results

### Genistein treatment reduces body weight gain in gonadectomized mice with DIO

**Figure 1:**
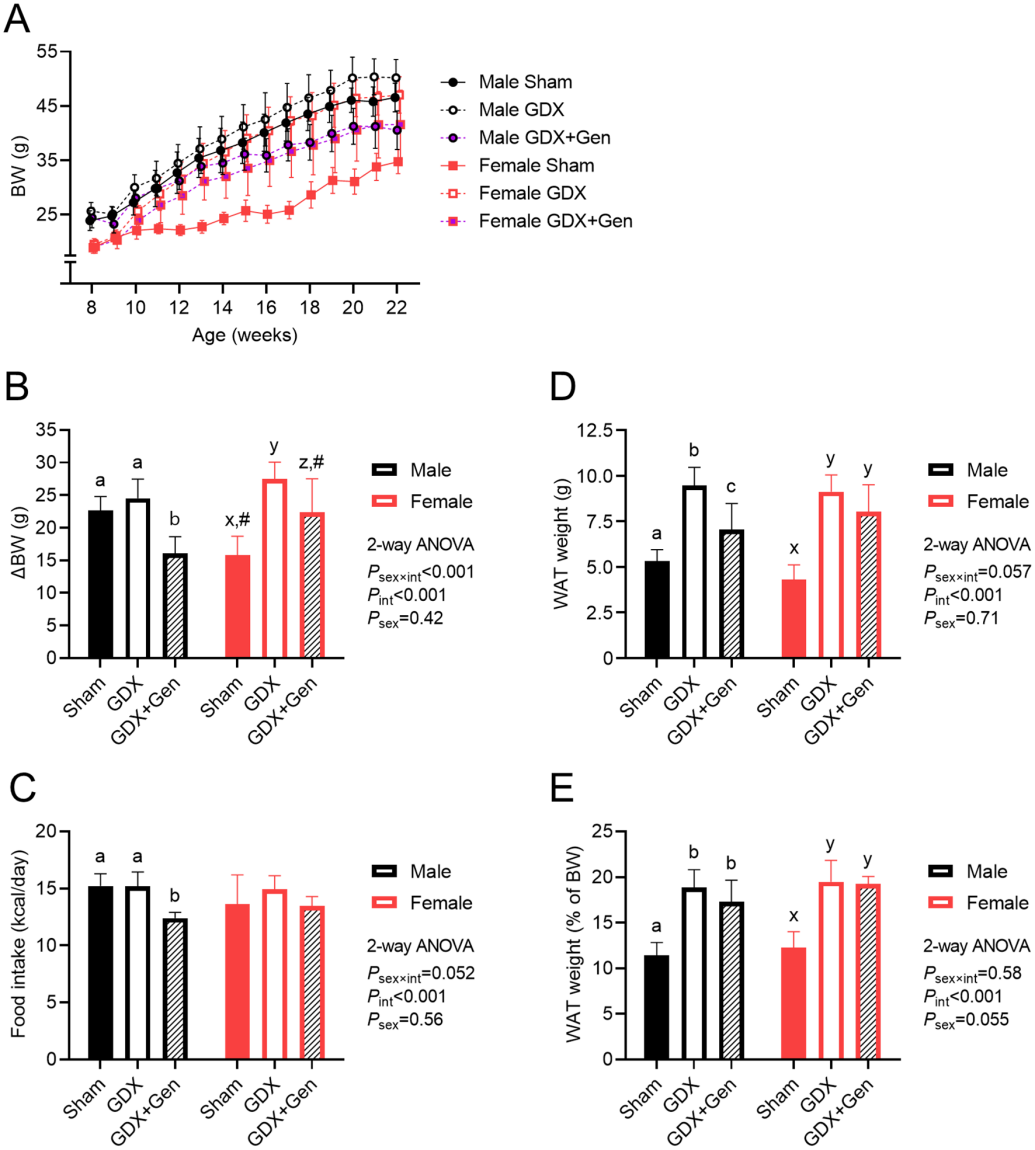
Effects of gonadectomy and genistein treatment on BW and energy balance in HFHS diet-fed mice. **(A)** BW of mice (n=7/group) across the experimental period: gonadectomy or sham-operation performed on week 8, *ad libitum* HFHS diet provided from weeks 9 to 22, and genistein treatment administered orally from weeks 13 to 22. **(B)** BW gain from weeks 8 to 22. **(C)** Estimated daily calorie intake, calculated based on food consumption from weeks 9 to 22. **(D)** Total WAT weight and **(E)** WAT weight relative to BW, measured at the end of the experiment. GDX (gonadectomized mice treated with vehicle), GDX+Gen (gonadectomized mice treated with genistein), Sham (sham-operated mice treated with vehicle). The statistical significance was determined by 2-way ANOVA for interventions (gonadectomy and genistein treatment) and sex (biological sex of the mice). The appropriate *post hoc* tests were conducted: letters a,b,c denote significant differences (*P*<0.05) among intervention groups of male mice; letters x,y denote significant differences (*P*<0.05) among intervention groups of female mice; and ^#^ indicates a significant sex difference (*P*<0.05) from male mice with the same intervention.

The longitudinal changes in BW of all mice across the experiment are demonstrated in **Figure 1A**. At the beginning of the experiment, female mice had lower BW than male mice, but within each sex, no significant differences were observed among the intervention groups (BW at age of 8 weeks: *P*_sex×int_=0.57, *P*_int_=0.12, *P*_sex_<0.001). By the end of the experiment upon DIO, BW was significantly influenced by interventions (gonadectomy and genistein treatment) and sex (BW at age of 22 weeks: *P*_sex×int_<0.001, *P*_int_<0.001, *P*_sex_<0.001). Notably, the change in BW from baseline to the end of the experiment revealed a significant interaction between the effects of interventions and sex (**Figure 1B**). In male mice, the BW gain did not significantly differ between the Sham and GDX groups, but the BW gain of the GDX+Gen group was significantly lower than that of the Sham and GDX groups (**Figure 1B**). Conversely, in female mice, the change in BW significantly varied across interventions; specifically, the BW gain in the Sham group was lower than that of the GDX group, and the BW gain of the GDX+Gen group was lower than that of the GDX group but remained higher than that of the Sham group (**Figure 1B**). When comparing between sexes, the BW gain of female Sham mice was significantly lower than that of male Sham mice, whereas the BW gain of female GDX+Gen mice was significantly higher than that of male GDX+Gen mice (**Figure 1B**).

In male mice, food consumption was lower in the GDX+Gen group compared to the Sham and GDX groups, while in female mice, food intake did not significantly differ among the three intervention groups (**Figure 1C**). Regression analysis in the GDX and GDX+Gen groups identified significant factors contributing to BW changes, including genistein treatment, biological sex, amount of food intake, fasting glucose and insulin levels, and the AUC of glucose after a glucose challenge (**Supplementary Table 2**). Further investigation into these parameters was conducted.

The total WAT weight was lowest in the Sham groups of both sexes, without a significant sex difference (**Figure 1D**). While the GDX groups exhibited a higher WAT mass than the Sham groups in both sexes, genistein treatment significantly reduced the WAT mass only in male mice (**Figure 1D**). When adjusted for BW, the ratio of WAT weight to BW was also lowest in the Sham groups and gonadectomy increased this ratio without a significant sex difference (**Figure 1E**). However, genistein treatment did not significantly affect the ratio of WAT weight to BW in both sexes (**Figure 1E**).

### Genistein treatment mitigates the impaired glucose metabolism

**Figure 2:**
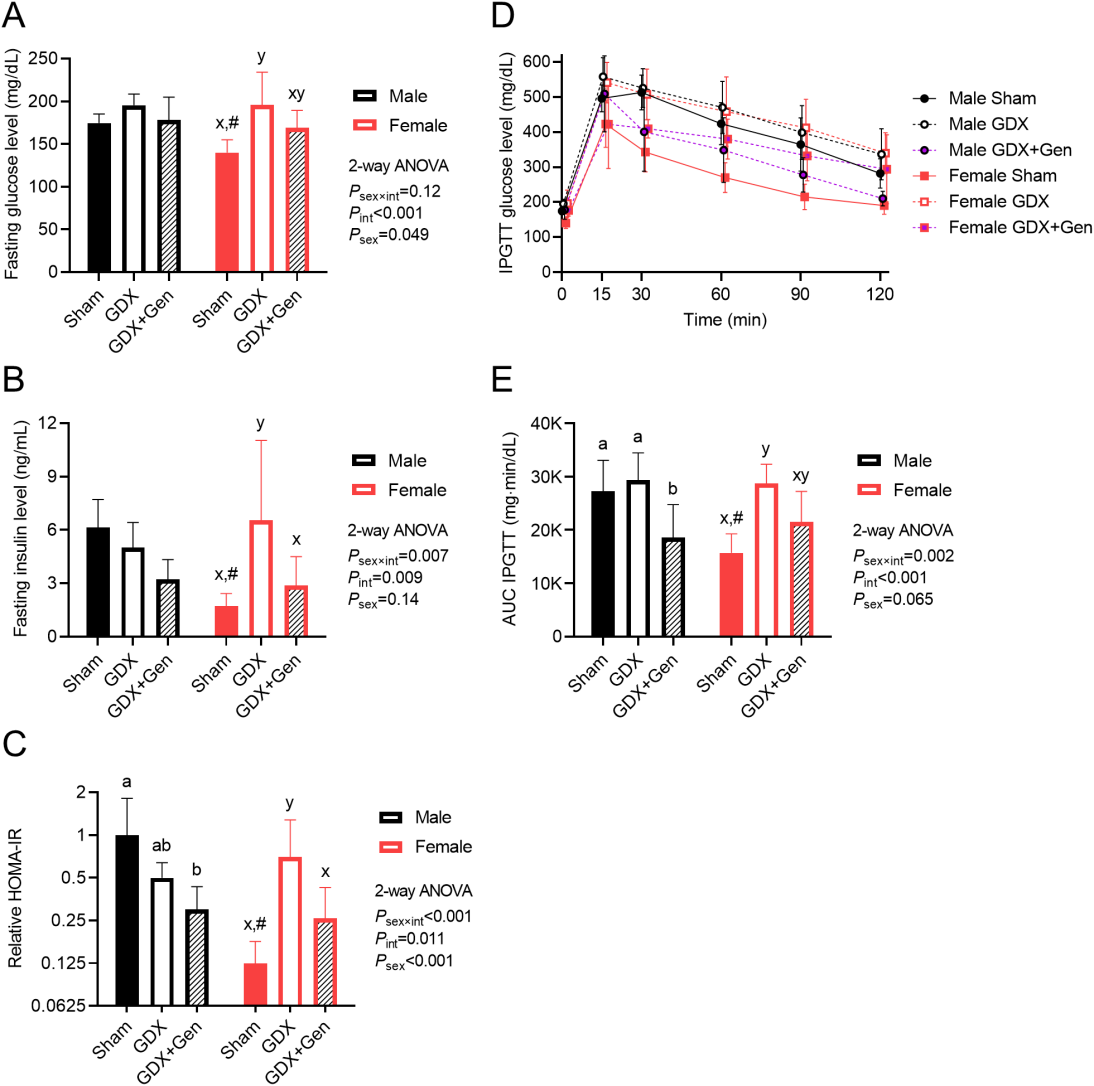
Effects of gonadectomy and genistein treatment on glucose homeostasis in HFHS diet-fed mice. **(A)** Blood glucose levels and **(B)** plasma insulin levels, determined after a 5-hour fasting period. **(C)** HOMA-IR indices calculated relative to the average value of the male sham-operated mice treated with vehicle and plotted on the logarithmic scale. **(D)** Blood glucose levels and **(E)** baseline-corrected AUCs of glucose levels after intraperitoneal glucose administration. GDX (gonadectomized mice treated with vehicle), GDX+Gen (gonadectomized mice treated with genistein), Sham (sham-operated mice treated with vehicle). The statistical significance was determined by 2-way ANOVA for interventions (gonadectomy and genistein treatment) and sex (biological sex of the mice). The appropriate *post hoc* tests were conducted: letters a,b denote significant differences (*P*<0.05) among intervention groups of male mice; letters x,y denote significant differences (*P*<0.05) among intervention groups of female mice; and ^#^ indicates a significant sex difference (*P*<0.05) from male mice with the same intervention.

Upon DIO, in male mice, fasting glucose levels did not significantly differ among the three intervention groups, while in female mice, those in the GDX group exhibited significantly higher levels than the Sham group (**Figure 2A**). Additionally, fasting glucose levels in female GDX+Gen mice tended to be lower than those of female GDX mice, though not statistically significant (*P*=0.081; **Figure 2A**). In male mice, fasting insulin levels did not significantly differ among the three intervention groups (**Figure 2B**). Conversely, in female mice, fasting insulin levels were significantly higher only in the GDX group, compared to the Sham and GDX+Gen groups (**Figure 2B**). The HOMA-IR index, a surrogate marker of IR, demonstrated a significant sex difference. In male mice, the HOMA-IR did not significantly differ between the Sham and GDX groups, but genistein treatment significantly lowered the HOMA-IR of the GDX+Gen group compared to the Sham group (**Figure 2C**). Contrarily, in female mice, the HOMA-IR was significantly higher in the GDX group than in the Sham group, and genistein treatment reduced the HOMA-IR of the GDX+Gen group to a level comparable with the Sham group (**Figure 2C**). In addition, when comparing between sexes, the female Sham group exhibited lower levels of fasting glucose, fasting insulin, and HOMA-IR than the male Sham group in this DIO model (**Figures 2A–C**).

Regarding IPGTT, a significant interaction between the effects of interventions and sex was observed (**Figures 2D, E**). In male mice, the change in glucose levels over time after the glucose challenge (quantification of glucose intolerance) did not significantly differ between the Sham and GDX groups. However, genistein treatment significantly reduced the AUC of glucose levels in the GDX+Gen group to a lower level than those in the other two groups (**Figure 2E**). In female mice, the AUC of glucose levels after IPGTT in the GDX group was significantly higher than that in the Sham group. Comparing the female GDX+Gen to GDX groups, genistein treatment tended to reduce glucose intolerance, though not statistically significant (*P*=0.053; **Figure 2E**). Of note, in this DIO model, female Sham mice exhibited lower glucose intolerance than male Sham mice (**Figures 2D, E**).

### Genistein treatment alleviates HFHS diet-induced steatohepatitis

**Figure 3:**
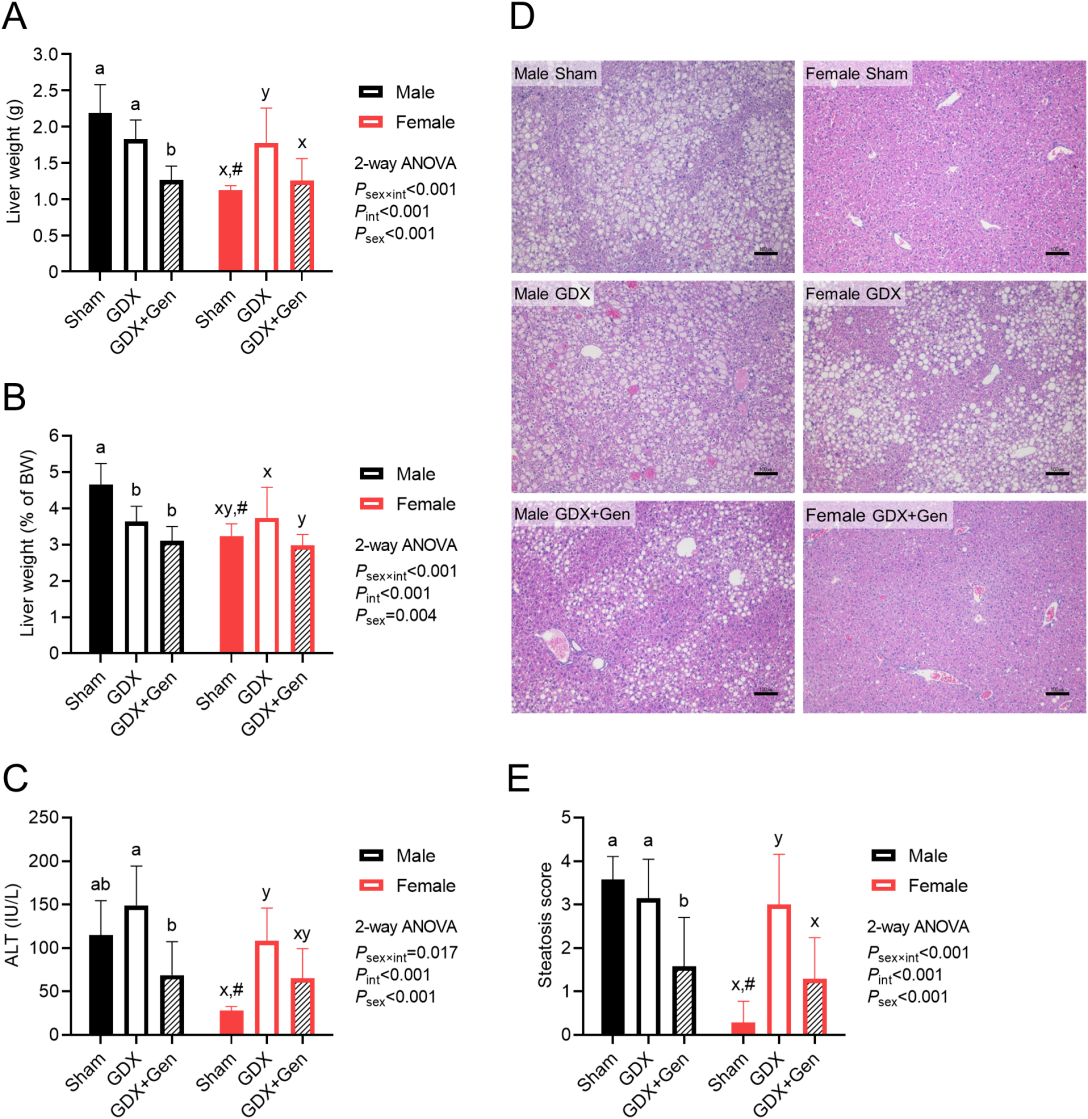
Effects of gonadectomy and genistein treatment on HFHS diet-induced steatohepatitis. **(A)** Liver weight, **(B)** liver weight relative to BW (the liver index) and **(C)** serum ALT levels, measured at the end of the experiment. **(D)** Representative images of hepatic histopathological findings, with scale bars in the lower right corners indicating 100 µm. **(E)** Histologic quantification of lipid accumulation in the liver. The statistical significance was determined by 2-way ANOVA for interventions (gonadectomy and genistein treatment) and sex (biological sex of the mice). The appropriate *post hoc* tests were conducted: letters a,b denote significant differences (*P*<0.05) among intervention groups of male mice; letters x,y denote significant differences (*P*<0.05) among intervention groups of female mice; and ^#^ indicates a significant sex difference (*P*<0.05) from male mice with the same intervention.

Upon HFHS diet-induced metabolic dysfunction, while orchiectomy (gonadectomy in males) tended to reduce the total liver weight and significantly reduced the liver index in male mice, ovariectomy (gonadectomy in females) significantly increased the total liver weight and tended to increase the liver index in female mice (**Figures 3A, B**). However, genistein treatment in the gonadectomized mice significantly reduced or tended to reduce the total liver weight and the liver index in both sexes (**Figures 3A, B**). Additionally, linear regression analysis identified significant factors contributing to the liver index in the GDX and GDX+Gen groups, including genistein treatment, changes in BW, non-fasting glucose levels, and the HOMA-IR (**Supplementary Table 2**).

Regarding serum ALT levels, which generally indicate hepatocellular injury, orchiectomy tended to increase serum ALT levels in male mice, while ovariectomy significantly increased serum ALT levels in female mice (**Figure 3C**). Although genistein treatment appeared to attenuate the elevation of serum ALT levels in both sexes, the reduction in ALT levels in the GDX+Gen groups, compared to the GDX groups, was statistically significant only in male mice (**Figure 3C**).

Orchiectomy did not induce fat accumulation in the liver of male GDX mice compared to male Sham mice, whereas ovariectomy significantly induced hepatic fat accumulation in female mice, confirmed by histopathological findings and the steatosis score (**Figures 3D, E**). However, genistein treatment significantly reduced hepatic fat accumulation in the GDX+Gen groups across both sexes (**Figures 3D, E**).

It is worth noting that female Sham mice exhibited lower features of MASLD in this DIO model than male Sham mice, as demonstrated by lower total liver weight, liver index, serum ALT, and hepatic steatotic score (**Figures 3A–E**). However, genistein treatment in the gonadectomized mice reduced these markers of MASLD comparably in both sexes (**Figures 3A–E**).

### Genistein treatment sex-differentially influences expression of hepatic genes associated with IR and MASLD progression

**Figure 4:**
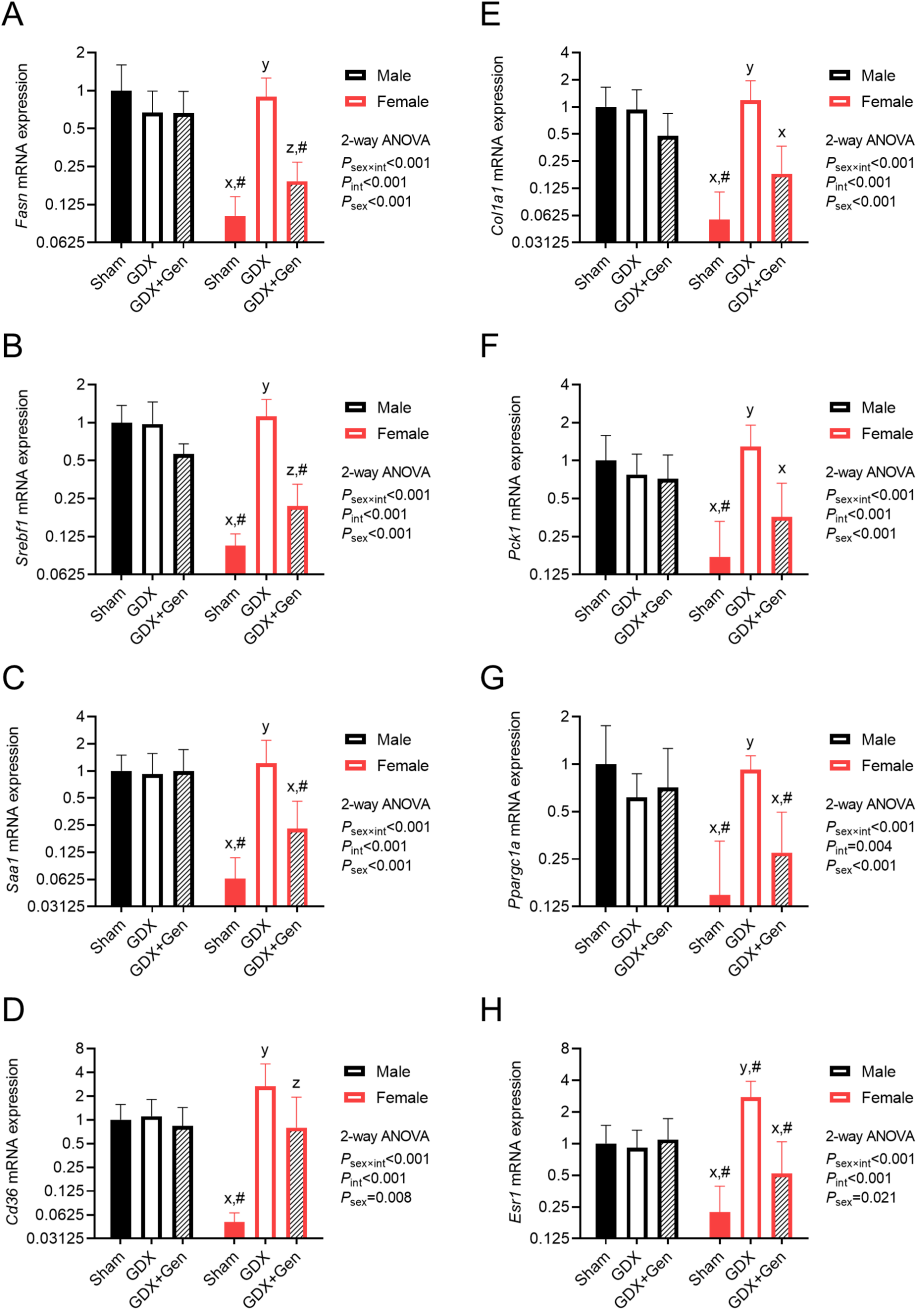
Effects of gonadectomy and genistein treatment on hepatic genes in HFHS diet-fed mice. Hepatic gene expression levels of **(A)** *Fasn*, **(B)** *Srebf1*, **(C)** *Saa1*, **(D)** *Cd36*, **(E)** *Col1a1*, **(F)** *Pck1*, **(G)** *Ppargc1a*, and **(H)** *Esr1*, normalized to *Rn18s* and *Actb* expression and presented on the logarithmic scale. The statistical significance was determined by 2-way ANOVA for interventions (gonadectomy and genistein treatment) and sex (biological sex of the mice). The appropriate *post hoc* tests were conducted: letters x,y,z denote significant differences (*P*<0.05) among intervention groups of female mice; and ^#^ indicates a significant sex difference (*P*<0.05) from male mice with the same intervention.

Upon HFHS diet feeding, significant interactions between the effects of interventions (gonadectomy and genistein treatment) and the sex of the mice were observed in the messenger RNA (mRNA) expression levels of hepatic genes involved in lipogenesis, MASLD progression, and gluconeogenesis. *Post hoc* analysis within groups of the same sex indicated that genistein treatment in the gonadectomized mice significantly reduced the expression of *Fasn*, the gene encoding fatty acid synthase (a key lipogenic enzyme), as well as its transcription factor *Srebf1*, exclusively in female mice (**Figures 4A, B**). Moreover, expression of *Saa1*, the gene encoding serum amyloid A1 which plays a role in inflammatory progression, was also reduced by genistein treatment only in female mice (**Figure 4C**). Likewise, the expression levels of *Cd36*, the gene encoding fatty acid translocase (a fatty acid transporter into hepatocytes), and *Col1a1*, the gene encoding type I collagen which plays a role in fibrotic progression, were significantly reduced only in female GDX+Gen mice, compared to female GDX mice (**Figures 4D, E**). In addition, expression levels of *Pck1*, the gene encoding cytosolic phosphoenolpyruvate carboxykinase (PEPCK, a rate-limiting gluconeogenic enzyme), as well as its transcriptional coactivator *Ppargc1a*, were significantly reduced by genistein treatment only in female mice (**Figures 4F, G**). However, in male mice, the expression of all these genes did not significantly differ between the GDX and GDX+Gen groups (**Figures 4A–G**), In other words, this hepatic gene expression pattern suggests the protective role of genistein treatment exclusively in ovariectomized mice.

Furthermore, within the same intervention groups, the expression of all the genes significantly differed between male and female Sham groups (**Figures 4A–G**). Intriguingly, while gonadectomy did not significantly affect the expression of these genes in male mice, it induced their expression in female GDX mice to a level comparable to that of male GDX mice (**Figures 4A-G**), suggesting sex-differential effects of endogenous gonadal hormones in regulating MASLD progression and IR.

To investigate whether estrogen receptor signaling is involved in the mechanism of action of genistein, we found that expression of *Esr1*, the gene encoding ERα, was significantly altered by gonadectomy and genistein treatment in female mice (**Figure 4H**). Of note, we could not detect mRNA expression of *Esr2*, the gene encoding ERβ, in the liver of both sexes.

## Discussion

This study confirms that genistein treatment improved impairments of glucose metabolism, such as HOMA-IR and glucose tolerance, as well as features of MASLD associated with HFHS diet-induced obesity and metabolic dysfunctions in both sexes of gonadectomized mice. However, our findings reveal some sex-differential effects of genistein treatment. Genistein treatment reduced food consumption only in orchiectomized mice. Conversely, genistein treatment decreased the expression of hepatic genes involved in the development and progression of MASLD and IR exclusively in ovariectomized mice. In other words, this study highlights the roles of endogenous gonadal hormones, particularly female sex hormones: the protective mechanisms against impaired glucose metabolism and MASLD features, as observed in sham-operated female mice, were diminished by ovariectomy.

Sex hormone deficiencies, including menopause and late-onset hypogonadism in men (so-called andropause), are associated with increased visceral fat accumulation and an accelerated risk of metabolic diseases. In our study, we employed a mouse model of HFHS diet-induced obesity to investigate obesity-related metabolic disturbances in the context of sex hormone deprivation. We observed that both male and female GDX mice had increased total WAT mass, but the increased BW was evident only in female GDX mice. The unchanged BW of male GDX mice, compared to male Sham mice, could be explained by the decline in muscle mass in contrast to increased adiposity due to testosterone deprivation (19). In our study, we observed that genistein treatment reduced food intake and fat accumulation in WAT exclusively in orchiectomized males. This finding contradicts the conventional understanding that estrogens exert anorexigenic effects, and ovariectomy promotes hyperphagia and induces adiposity in females (20). However, we could not find direct evidence investigating the effects of genistein on food intake and fat accumulation in orchiectomized males. Previous research on ovariectomized rats also confirmed the appetite-suppressing effect of soybean isoflavone by altering orexigenic signals (hunger hormones) and anorexigenic signals (satiety hormones) (21). Our findings in female mice partially align with a previous study in ovariectomized rats, which found that genistein administration at doses of 15 or 30 mg/kg/day did not alter food intake, but dose-dependently reduced fat accumulation in WAT (22). Thus, differences in genistein dosage, treatment duration, or the method used to induce obesity may contribute to the observed discrepancies.

Sex hormone deprivation is also associated with metabolic derangements, impacting lipid and glucose metabolism. Estrogen has been identified as a protective factor against metabolic syndrome, including IR and MASLD. Lack of estrogen in females exacerbates IR and hepatic steatohepatitis in rodent models of DIO (23, 24). Consistent with previous findings, our study showed that female GDX mice exhibited significantly more severe IR and hepatic steatohepatitis than female Sham mice. While androgen in males may not play as prominent a protective role as estrogen in females, androgen deficiency in males has been associated with metabolic syndrome. Research on orchiectomized mice has shown that androgen deficiency aggravates high-fat diet-induced metabolic derangements, including increased adiposity and IR (19). Interestingly, the metabolic derangements in male hypogonadism were restored by testosterone but not dihydrotestosterone supplementation (19), suggesting that the beneficial effect of testosterone may be achieved through aromatization to estradiol. However, our findings that IR and hepatic steatohepatitis in male GDX mice did not significantly differ from male Sham mice did not align with previous evidence and warrant further studies.

In our study, genistein treatment significantly attenuated impairment of glucose metabolism, as represented by HOMA-IR, and steatohepatitis in female GDX mice. These findings are consistent with evidence from previous studies demonstrating that genistein treatment reduces fat accumulation in the liver and alleviates hepatic steatosis in postmenopausal women or ovariectomized rodents (13, 25), as well as effectively reducing IR in estrogen-deprived female rodents and women (26, 27). Additionally, recent studies in female mice reported that genistein treatment significantly improved IR or glucose intolerance in mice with DIO by various mechanisms such as promoting hepatic microRNA-451 expression, reducing proinflammatory cytokine production in WAT, inducing WAT browning, and modulating gut microbiota (22, 28, 29). However, the effect of genistein on androgen-deprived males has been limited. Our findings indicated that male GDX+Gen mice exhibited significantly decreased serum ALT levels and liver steatosis compared to male GDX mice. Additionally, a recent study from our group also supported our present findings that genistein treatment in orchiectomized rats alleviated hepatic steatohepatitis, as evident by suppression of NF-ĸB expression and upregulation of HDAC3 and PPARδ expression (30). Thus, our results highlighted the beneficial effects of genistein on impaired glucose metabolism and MASLD in estrogen-deprived females and suggested that genistein may also be beneficial in androgen-deprived males.

The expression levels of selected genes investigated in our study serve as reliable markers for MASLD progression and hepatic IR. Hepatic mRNA expression of FASN, the enzyme for *de novo* lipogenesis, and its transcription factor [sterol regulatory element-binding protein-1c (SREBP-1c)], has been reported to be upregulated in MASLD, but not in metabolic dysfunction-associated steatohepatitis (MASH) in murine models (31, 32). Increased SREBP-1c expression has been demonstrated to be associated with high-fat or high-fructose diet feeding, high insulin levels or IR state, obesity, and MASLD (33). Interestingly, its expression was reduced by soy protein or genistein supplementation (34, 35), suggesting a protective mechanism of genistein against MASLD progression. Fatty acid translocase (CD36) is not only a fatty acid transporter into hepatocytes but also a potential marker of MASLD onset and progression to MASH (36). A previous study using a high-fat diet to induce obesity in mice demonstrated, in line with our finding, that genistein treatment reduced *Cd36* mRNA expression (22, 37). We also examined the expression of *Col1a1* as a fibrosis marker and *Saa1* as an inflammatory marker since they have been reported to be highly correlated with advanced stages of MASLD (38, 39). Although the histopathological findings in our study did not show significant alterations in hepatic inflammation and fibrosis, diminished levels of *Col1a1* and *Saa1* mRNAs suggest a potential role of genistein treatment in slowing MASLD progression. Additionally, we evaluated mRNA expression levels of PEPCK, a rate-limiting enzyme for hepatic gluconeogenesis, and its transcription factor coactivator PGC1α, as their expression is physiologically inhibited by insulin in the fed state, and aberrant activation of gluconeogenesis indicates hepatic IR (40, 41).

In our study, significant interactions between the effects of interventions (gonadectomy and genistein treatment) and sex were observed on the expression levels of all investigated hepatic genes. Female mice with HFHS diet-induced obesity initially exhibited low expression levels of these genes, which significantly increased upon ovariectomy to levels comparable to those in male mice. This pattern of changes in hepatic gene expression supports previous studies demonstrating a lower prevalence of MASLD in premenopausal women compared to men, with an accelerated prevalence in post-menopausal women (42). Our study also showed that genistein treatment reduced the expression of these hepatic genes in ovariectomized mice, supporting previous findings that genistein treatment inhibited the expression of genes involved in lipogenesis (*Fasn*, *Srebf1*, *Cd36*) and gluconeogenesis (*Pck1*) in the liver of female rodents with DIO (22, 43). Nonetheless, we did not observe a significant effect of genistein treatment on the expression of these hepatic genes in orchiectomized mice. Importantly, our study is one of the first experiments to directly investigate the effects of genistein in both sexes and reveals significant sex differences in MASLD-related gene expression profiles in the liver.

There were some limitations in our study. We did not vary the administered dose of genistein to explore its potential dose-dependent effects, and serum genistein levels were not measured due to the unavailability of the required instruments. The dosage of genistein used in our study has been shown to be beneficial in prior research, including our own (13, 16). The oral dose of genistein at 16 mg/kg/day in mice was likely equivalent to the dose of 78 mg/day in 60-kilogram adults (17), with a daily dose of 36–600 mg suggested as beneficial in various human studies (44). Furthermore, our findings preliminarily indicated sex differences in hepatic gene expression related to lipogenicity, inflammation, fibrosis, and IR, although no significant sex differences were observed in hepatic steatosis by histopathology or serum ALT levels. A possible explanation could be the insensitivity of serum ALT for stratifying MASLD progression and the high variability in its progression (45). Hence, more sensitive and specific measures, such as immunohistochemical staining, may be required in further studies. Additionally, it is important to note that our model of sex hormone deprivation, achieved through surgical removal of gonads, combined with DIO may not precisely replicate the metabolic changes in individuals with obesity and sex hormone deprivation due to aging.

## Conclusion

Genistein treatment mitigates obesity-related metabolic dysfunctions, particularly impaired glucose metabolism and MASLD, in both male and female GDX mice with HFHS diet-induced obesity. The protective effect of genistein treatment, specifically in terms of the suppression of genes related to MASLD progression, was evident exclusively in female GDX mice. Pending further validation through clinical trials, this finding suggests a potential alternative application for the natural phytoestrogen, genistein, as a dietary supplement for both men and women dealing with obesity and sex hormone deprivation, such as those in the aging population.

## Data Availability

The original contributions presented in the study are included in the article/supplementary material. Further inquiries can be directed to the corresponding author.
