# Supplementary material for: Genistein mitigates diet-induced obesity and metabolic dysfunctions in gonadectomized mice with some sex-differential effects
Source: Front Endocrinol (Lausanne). 2024 Sep 16;15:1392866. doi: 10.3389/fendo.2024.1392866 (PMC11439649; doi:10.3389/fendo.2024.1392866)
Supplement: Supplementary file 1 [file Table1.docx]

**Supplementary Table 1** Primer sequences for mRNA expression quantification

| **Genes** | **Forward primer sequence** | **Reverse primer sequence** |
| --- | --- | --- |
| *Actb* | ACACCCGCCACCAGTTC | TACAGCCCGGGGAGCAT |
| *Cd36* | CCTCCAGAATCCAGACAACC | CACAGGCTTTCCTTCTTTGC |
| *Col1a1* | GCTCCTCTTAGGGGCCACT | CCACGTCTCACCATTGGGG |
| *Esr1* | TGGGTCATGAGAGTCCTTTGAA | CCGGGATGGAAACTGAACTTT |
| *Esr2* | TTCTTTCTCATGTCAGGCACA | CTCGAAGCGTGTGAGCATT |
| *Fasn* | GCTGCTGTTGGAAGTCAGC | AGTGTTCGTTCCTCGGAGTG |
| *Pck1* | AAGTGCCTGCACTCTGTGG | CAGGCCCAGTTGTTGACC |
| *Ppargc1a* | CCCTGCCATTGTTAAGACC | TGCTGCTGTTCCTGTTTTC |
| *Rn18s* | GTAACCCGTTGAACCCCATT | CCATCCAATCGGTAGTAGCG |
| *Saa1* | CTGGTCTTCTGCTCCCTGC | TTGTCTGAGTTTTTCCAGTTAGC |
| *Srebf1* | GGAGCCATGGATTGCACATT | CCTGTCTCACCCCCAGCATA |
