# Supplementary material for: Genistein mitigates diet-induced obesity and metabolic dysfunctions in gonadectomized mice with some sex-differential effects
Source: Front Endocrinol (Lausanne). 2024 Sep 16;15:1392866. doi: 10.3389/fendo.2024.1392866 (PMC11439649; doi:10.3389/fendo.2024.1392866)
Supplement: Supplementary file 2 [file Table2.docx]

**Supplementary Table 2** Factors contributing to the change in BW and the liver index (the ratio of liver weight to BW) of mice in the GDX and GDX+Gen groups

| **Factors** | **BW change** | | **Liver index** | |
| --- | --- | --- | --- | --- |
|  | **Standardized β** | ***P* value** | **Standardized β** | ***P* value** |
| Genistein treatment | 0.22 | **<0.001** | −0.33 | **<0.001** |
| Sex | 0.28 | **<0.001** | NA | 0.53 |
| Initial BW | NA | 0.95 | NA | 0.27 |
| Food intake | 0.50 | **<0.001** | NA | 0.11 |
| BW change | – | – | −0.61 | **<0.001** |
| Non-fasting glucose level | NA | 0.17 | −0.29 | **0.012** |
| Fasting glucose level | 0.17 | **0.015** | NA | 0.90 |
| Fasting insulin level | 0.15 | **0.039** | NA | 0.29 |
| HOMA-IR | NA | 0.80 | 0.93 | **<0.001** |
| Baseline-corrected AUC of glucose levels in IPGTT | 0.31 | **<0.001** | NA | 0.59 |

Statistically significant factors in stepwise linear regression analysis are marked in bold. Abbreviations: AUC (area under the curve), BW (body weight), GDX (gonadectomized mice treated with vehicle), GDX+Gen (gonadectomized mice treated with genistein), HOMA-IR (homeostatic model assessment for insulin resistance), IPGTT (intraperitoneal glucose tolerance test), and NA (not applicable).
